# Use of a novel cell-based fusion reporter assay to explore the host range of human respiratory syncytial virus F protein

**DOI:** 10.1186/1743-422X-2-54

**Published:** 2005-07-13

**Authors:** Patrick J Branigan, Changbao Liu, Nicole D Day, Lester L Gutshall, Robert T Sarisky, Alfred M Del Vecchio

**Affiliations:** 1 Infectious Diseases Research, Centocor, Inc., 145 King of Prussia Road, Radnor, PA, 19087, USA

## Abstract

Human respiratory syncytial virus (HRSV) is an important respiratory pathogen primarily affecting infants, young children, transplant recipients and the elderly. The F protein is the only virion envelope protein necessary and sufficient for virus replication and fusion of the viral envelope membrane with the target host cell. During natural infection, HRSV replication is limited to respiratory epithelial cells with disseminated infection rarely, if ever, occurring even in immunocompromised patients. However, in vitro infection of multiple human and non-human cell types other than those of pulmonary tract origin has been reported. To better define host cell surface molecules that mediate viral entry and dissect the factors controlling permissivity for HRSV, we explored the host range of HRSV F protein mediated fusion. Using a novel recombinant reporter gene based fusion assay, HRSV F protein was shown to mediate fusion with cells derived from a wide range of vertebrate species including human, feline, equine, canine, bat, rodent, avian, porcine and even amphibian (*Xenopus*). That finding was extended using a recombinant HRSV engineered to express green fluorescent protein (GFP), to confirm that viral mRNA expression is limited in several cell types. These findings suggest that HRSV F protein interacts with either highly conserved host cell surface molecules or can use multiple mechanisms to enter cells, and that the primary determinants of HRSV host range are at steps post-entry.

## Background

Human respiratory syncytial virus (HRSV) is the single most common cause of serious lower respiratory tract infections (LRTIs) in infants and young children causing up to 126,000 hospitalizations annually in the U.S. [1] with an estimated 500 deaths per year [2]. HRSV bronchiolitis has been associated with the development and exacerbation of wheezing and other respiratory conditions. Furthermore, HRSV is an increasingly recognized cause of pneumonia, exacerbation of chronic pulmonary and cardiac disease, and death in the elderly [3]. HRSV is also the most common viral respiratory infection of transplant recipients and is responsible for high rates of mortality in this group [4].

HRSV is member of the subfamily *Pneumovirinae *in the *Paramyxoviridae *family [5]. Two serologic subgroups (A and B) have been described and co-circulate throughout the world. Three viral transmembrane proteins (G, SH and F) are found on surface of the virus particle in the viral envelope. The G protein is a heavily glycosylated protein that mediates attachment of the virus to host cells, and although not strictly required for virus replication in culture, recombinant viruses lacking the G protein are attenuated in animals [6,7]. While its exact function is unknown, the SH gene is not essential for virus growth in tissue culture, and its deletion only results in slight attenuation in animals [6,8-12]. The F protein is a type 1 membrane protein essential for the packaging and formation of infectious virion particles [6,7,13,14], and is the only viral protein necessary and sufficient for fusion of the viral envelope membrane with the target host cell [15,16]. The HRSV F protein is highly conserved (89% amino acid identity) between subgroups A and B, and shares 81% amino acid identity with the F protein of bovine respiratory syncytial virus (BRSV). The HRSV F protein is synthesized as a 574 amino acid precursor protein designated F0, which is cleaved at two sites [17-20] within the lumen of the endoplasmic reticulum removing a short, glycosylated intervening sequence and generating two subunits designated F1 and F2 [21]. The mature form of the F protein present on the surface of the virus and infected cells is believed to consist of a homotrimer consisting of three non-covalently associated units of F1 disulfide linked to F2[22]. As with many other viral fusion proteins, F-mediated fusion with the host cell membrane is believed to be mediated by insertion of a hydrophobic fusion peptide into the host cytoplasmic membrane after binding of F protein to a target receptor on the host cell. Subsequent conformational changes within F result in the interaction of heptad repeat (HR) 1 with HR2 and formation of a 6-helix bundle structure [22-24]. This process brings the viral and host cell membranes in close proximity resulting in fusion pore formation, lipid mixing, and fusion of the two membranes. The precise number of F trimers and identity of target host surface proteins or molecules required to mediate fusion are currently unknown.

Although initially isolated from a chimpanzee, humans are the primary natural host for HRSV. HRSV will only infect the apical surface of human ciliated lung epithelial cells, and only fully differentiated human bronchial epithelial cells are permissive for HRSV growth [25]. Dissemination of HRSV to other organs is not observed even in immunocompromised individuals. Similarly, disseminated infection with bovine RSV is not observed in infected cattle. In contrast, in vitro infection of multiple human cell types other than those derived from lung [26], cells derived from other animal species, and HRSV infection of several animal species has been reported [27]. This suggests that the F protein interacts with either highly conserved host cell surface molecules or can use multiple mechanisms to mediate fusion. Several previous studies have shown the importance of cell-surface glycosaminoglycans (GAGs) [28-32], in particular iduronic acid, in mediating HRSV infection in vitro [33]; however, GAG independent, F-mediated attachment pathways have been described [13]. In a study comparing the host range of bovine and human respiratory syncytial viruses for cells derived from their respective natural hosts, species specificity mapped to the F2 subunit [10]. These finding allude to the existence of host specific receptor molecules that specifically interact with the F protein to mediate cell fusion.

To better understand the factors governing host range, we developed a HRSV F-based quantitative cell fusion assay and specifically examined the ability of HRSV F protein to mediate fusion with cells derived from a wide range of animal species. As cell permissiveness for virus growth is dependent upon multiple steps, we went on to further characterize the permissiveness of these cells for viral mRNA transcription by using a recombinant HRSV engineered to express GFP [33]. The relevance of these findings to the natural course of HRSV disease is discussed.

## Methods

### Cells and viruses

All cell lines were obtained from the American Type Culture Collection (ATCC) (Manassas, VA) and were grown at 37°C in a humidified atmosphere of 5% CO_2 _with the exception of XLK-WG (grown at 32°C). BHK-21, E. Derm, HeLa, HEp-2, LLC-PK1, MDBK, MDCK, Mv1Lu, RK-13, Tb1Lu, Vero and A549 cells (ATCC CCL-10, CCL-57, CCL-2, CCL-23, CL-101, CCL-22, CCL-34, CCL-64, CCL-37, CCL-88, CCL-81, and CCL-185 respectively) were maintained in modified Eagle media (MEM) with 2 mM L-glutamine and Earle's balanced salt solution (BSS) adjusted to contain 1.5 g/L sodium bicarbonate, 0.1 mM non-essential amino acids, 1.0 mM sodium pyruvate and 10% heat-inactivated, gamma-irradiated fetal bovine serum (FBS) (HyClone Laboratories, Salt Lake City, Utah). AK-D cells (CCL-150) were maintained in Ham's F-12K media containing 10% FBS. NCI-H292 (CRL-1848), MT-4 and XLK-WG (CRL-2527) cells were maintained in RPMI 1640 medium with 2 mM L-glutamine adjusted to contain 1.5 g/L sodium bicarbonate, 4.5 g/L glucose, 10 mM HEPES, 1.0 mM sodium pyruvate and 10% FBS. NIH/3T3, QT6 and 293T cells (CRL-1658, CRL-1708, and CRL-1573) were maintained in Dulbecco's modified Eagle media (DMEM) with 4 mM L-glutamine adjusted to contain 1.5 g/L sodium bicarbonate, 4.5 g/L glucose and 10% FBS. Cell lines were maintained at sub-confluence and used for only up to 15 passages after receiving initial stocks from the ATCC. Cell lines were tested and confirmed to be free of mycoplasma contamination. Human RSV (subgroup A, Long strain, ATCC VR-26) was obtained from the ATCC. Virus stocks were prepared by infecting HEp-2 cells with RSV at a multiplicity of infection (MOI) of 0.01 plaque-forming units (PFU) per cell. When cytopathic effect (CPE) was evident (~6 days post infection), the culture supernatant was collected and pooled with the supernatant from the infected cell pellet, which had been subjected to one freeze-thaw cycle. The pooled supernatants were maintained on ice, adjusted to 10% sucrose, flash frozen in liquid nitrogen and stored in liquid nitrogen. RSV titers were determined by plaque assay on HEp-2 cells. Serial dilutions of virus stock were added to monolayers of HEp-2 cells at 80% confluence and allowed to adsorb for 2 hours at 37°C. The virus inoculum was then removed, and cells were overlayed with media containing 0.5% methylcellulose. After plaques became apparent (5–6 days after infection), cell monolayers were fixed and stained with 0.5% crystal violet in 70% methanol, and plaques were counted. HRSV stock titers were typically >10^6 ^PFU/ml and remained stable for 6 months without loss of titer. A recombinant RSV rgRSV(224) engineered to express GFP has been previously described [33]. Stocks of rgRSV(224) were prepared as described above. Cell lines were infected with rgRSV(224) at a MOI of 0.1 and infection was visualized by fluorescent microscopy by monitoring fluorescence at 488 nm at 20, 48, and 120 hours post infection.

### Plasmids

A DNA fragment encoding HRSV F protein derived from a known infectious cDNA sequence for subgroup A, A2 strain, [34] was synthesized with optimal codon usage for expression in mammalian cells and all potential polyadenylation sites (AATAAA) and splice donor sites (AGGT) removed essentially as described [15]. A similar construct was designed and synthesized for the B subgroup F protein (18537 strain, based upon GenBank accession number D00344). Sequence data is available from the authors upon request. Restriction sites for *Xba*I and *Bam*HI were added to the 5' and 3' ends of the fragments respectively. The codon optimized HRSV-F DNA fragments (A2 and 18537 strains) were then cloned into the *Xba*I and *Bam*HI sites of pcDNA 3.1 (Invitrogen, Inc., Carlsbad, CA) to generate pHRSVFOptA2 and pHRSVFOpt18537. The QuikChange^® ^Site-Directed Mutagenesis kit (Stratagene^®^, La Jolla, CA) was used to change leucine 138 in the fusion peptide region of the F protein to an arginine (pL138R) in pHRSVFOptA2. Plasmids pBD-NFκB encoding the activation domain of NFκB fused to the GAL4 DNA binding domain under the control of the human cytomegalovirus (HCMV) promoter and pFR-Luc containing the luciferase reporter gene under the control of a minimal promoter containing five GAL4 DNA binding sites were obtained from Stratagene^®^. pGL3-control vector encodes a modified firefly luciferase under the control of the SV40 early promoter (Promega, Inc.). Plasmid pVPack-VSV-G encodes the G protein of vesicular stomatitis virus (Stratagene^®^, La Jolla, CA).

### Transfections

Cells were transiently transfected using FuGENE 6 reagent (Roche Applied Science, IN) according to the manufacturer's recommendations. Briefly, 7.5 × 10^5 ^cells were plated in 6-well plates and grown overnight to ~90% confluence. Two micrograms of plasmid DNA was complexed with 6 μl of FuGENE 6 reagent for 30 minutes at room temperature in 100 μl of serum-free medium. The complex was then added to the cells and incubated at 37°C for 20–24 hours.

### Metabolic labeling and immunoprecipitation

293T cells were plated the day before transfection in 6-well plates at a density of 0.75 × 10^6 ^cells/well in DMEM supplemented with 10% FBS. Cells in 6-well plates were transfected with a total of 2 μgs of plasmid DNA as described above. At 20 hours post-transfection, cells were starved by incubation in DMEM without L-methionine and L-cysteine containing 5% dialyzed FBS for 45 minutes. Cells were then labeled by incubation in DMEM without L-methionine and L-cysteine containing 5% dialyzed FBS and Redivue Pro-mix *in vitro *cell labeling mix containing (100 μCi/ml, 1.5 mls./well) [^35^S]-methionine and [^35^S]-cysteine (Amersham Biosciences, Piscataway, New Jersey) for 4 hrs. Media was removed, and cells were harvested and washed in 1 ml 1X phosphate-buffered saline (PBS) and then lysed with 0.5 mls. of lysis buffer (50 mM Tris-HCl pH 7.5, 150 mM NaCl, 0.5% sodium deoxycholate, 1% IGEPAL (Sigma, St. Louis, MO) and Complete ™ protease inhibitor cocktail with EDTA (Roche Biochemicals, Indianapolis, IN). Lysates were spun for 30 minutes at 4°C to remove insoluble material and immunoprecipitated by incubation with a saturating amount (as determined by prior titration) of a cocktail containing 1.5 μgs of anti-F mAbs and protein-A agarose beads (Invitrogen, Inc., Carlsbad CA) overnight at 4?C. Immunoprecipitated complexes were washed three times in lysis buffer and suspended in 20 μl of 4X LDS NuPage loading buffer with reducing agent and resolved by electrophoresis through SDS-containing polyacrylamide gels (SDS-PAGE) under reducing conditions on a NuPage 4–12% Bis-Tris polyacrylamide gel (Invitrogen, Inc., Carlsbad CA). Gels were dried under vacuum for one hour at 80°C followed by autoradiography.

### Flow cytometry

To confirm cell surface expression of HRSV F, 293T cells were transfected in 6-well plates as described above for metabolic labeling. Cells were stained with palivizumab (Synagis ^®^, IgG1κ) at 1 μg/ml in conjunction with an anti-human IgG-Alexa-Fluor-488 conjugated secondary (Molecular Probes, Eugene, OR) at 1 μg/ml in 1X PBS with 2% FBS for analysis with the FACSCalibur (BD Bisociences, CA) by setting a live cell gate in the FSC/SSC plot and determining the mean fluorescence intensity in the FL1 channel. Data analysis was performed with Cell Quest and FloJo Analysis Software.

### Cell fusion assays

293T cells were co-transfected with pHRSVFOptA2 and pBD-NFκB (effectors cells), and the panel of cell lines from a variety of different species were transfected using FuGene-6 (Roche Biochemicals, Inc.) with the pFR-Luc reporter plasmid (reporter cells) using conditions described above. Alternatively, 293T cells were co-transfected with pHRSVFOptA2 and pFR-Luc, and the panel of cell lines from a variety of different species were transfected with the pBD-NFκB reporter plasmid. A plasmid encoding the vesicular stomatitis virus (VSV) G protein linked to the HCMV promoter (pVPack-VSV-G) was used as a positive viral fusion protein control. At 24 hours post transfection, unless otherwise specified, 3 × 10^4 ^of the transfected 293T (effector cells) were mixed with an equal amount of the various other transfected cells (target cells) in a 96-well plate and incubated an additional 24 hours prior to measurement of luciferase activity using the Steady Glo Luciferase reporter system (Promega, Inc.). The various cell lines were transfected with pGL3-control to determine relative differences in transfection efficiencies and cell-type specific expression of luciferase. To further normalize, a single preparation of effector cells was used per each experiment on the various reporter cell preparations. Cells individually transfected with the F expression plasmids, pBD-NFκB or pFR-Luc individually were used as negative controls.

## Results

### Expression of RSV F protein

**Figure 1:**
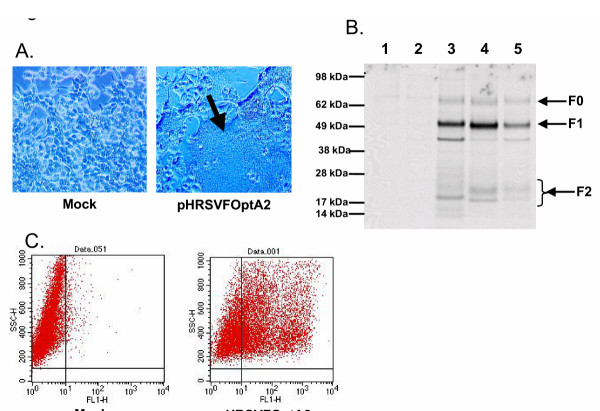
A) Syncytia formation by RSV-F DNA in transfected cells. 293T cells were mock transfected or transfected with pHRSVFOptA2 and visualized by light microscopy 48 hours post transfection. The arrow indicates giant multinucleated cell formation. B) Processing of RSV F in transfected cells. 293T cells were either mock transfected (lane 1), transfected with pCMV-β-gal (lane 2), transfected with pHRSVFOptA2 (lane 3), pHRSVFOptB18537 (lane 4), or infected with RSV (Long strain, MOI = 1) for 24 hours followed by metabolic labeling for 6 hours with [^35^S]-cysteine/methionine. Labeled cell lysates were immunoprecipitated with HRSV F specific mAbs, and immunoprecipitates were resolved by SDS-PAGE as described in methods. C) Cell surface expression of RSV F in transfected cells. 293T cells were either mock transfected or transfected with pHRSVFOptA2 for 24 hours followed by flow cytometry using HRSV F specific monoclonal antibodies as described in methods.

**Figure 2:**
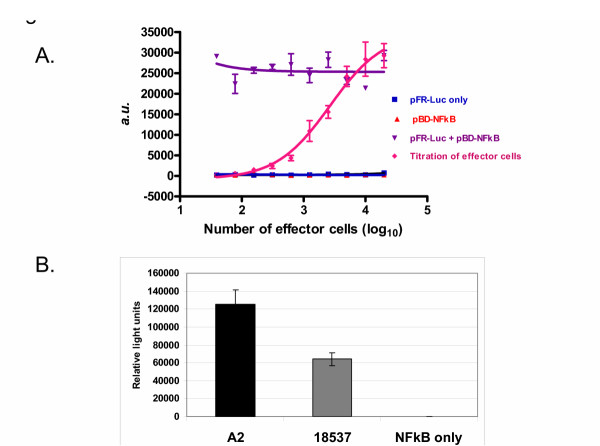
A) Dose dependent fusion activity of HRSV F derived. 293T cells were transfected with either pFR-Luc alone (■), pBD-NFκB alone (▲), co-transfected with pFR-Luc and pBD-NFκB (▼), or co-transfected with pHRSVFOptA2 and pBD-NFκB and mixed 24 hours after transfection in various amounts with cells that had been transfected with pFR-Luc alone (◆). Luciferase activity was measured 24 hours post mixing as described in methods and is reported as relative light units. B) Fusion activity of HRSV F derived from subgroups A and B. 293T cells co-transfected with pBD-NFκB and either pHRSVFOptA2, pHRSVFOptB18537, or vector only (NFκB only). Cells were mixed 24 hours later with a separate population of 293T cells transfected with pFR-Luc, and luciferase activity was measured 24 hours post mixing as described in methods. Luciferase activity is reported as relative light units.

We designed and synthesized a cassette encoding the full-length HRSV-F gene (A2 strain and 18537 strains) in which codon usage was optimized for mammalian expression and all potential splice-donor sites and polyadenylation sites were removed similar to a previous report [15]. Upon transient transfection of 293T cells with this plasmid expressing this optimized HRSV F protein sequence under the control of a human cytomegalovirus immediate early promoter (pHRSVFoptA2), giant multinucleated cells (syncytia) were readily apparent within 24 hours post transfection (Figure 1A). The amount of syncytia qualitatively increased throughout the culture for up to 72 hours, after which extensive cell death and sloughing was observed. This syncytia is phenotypically indistiguishable from that observed following infection of 293T cells with HRSV in tissue culture (data not shown). To confirm appropriate processing of the F protein, 293T cells were transfected with plasmids expressing HRSV F derived from subgroup A (A2 strain) or subgroup B (18537 strain) and metabolically labeled followed by immunoprecipitation of lysates with HRSV F-specific monoclonal antibodies. As a control, 293T cells were infected with HRSV (Long strain). As shown in Figure 1B, immunoprecipitation demonstrates the presence of the unprocessed full length F_0 _species migrating at approximately 70 kDa, and the processed F_1 _and F_2 _fragments of ~50 kDa and 20 kDa, respectively, identical to the F protein immunoprecipitated from HRSV infected 293T cells. The multiple bands observed in the region of 20 kDa likely represent the incompletely processed F2 (F2+), different glycosylated forms of F2, or a combination of both [21]. The band present migrating more rapidly than F1 (~35 kDa) most likely represents a cellular protein as this band was observed in lysates derived from untransfected and control vector transfected cells with varying intensity unrelated to the level of HRSV F expressed (see Figure 2B, lanes 3 and 4). Similar levels of expression were observed for the HRSV F protein from the A and B subgroups (Figure 1B, lane 3 compared to lane 4). Furthermore, the level of F protein expression in the transfected cells was greater at 24 hours post transfection than in HRSV-infected cells at 24 hours post infection (Figure 1B, lane 5). Flow cytometry confirmed abundant cell surface expression of HRSV F protein (Figure 1C).

### HRSV F protein fusion assay

To measure the ability of the HRSV F protein to mediate cell fusion across various cell lines, we developed a quantitative fusion assay. Specifically, 293T cells were co-transfected with plasmids encoding the optimized HRSV F protein and a transcriptional transactivator fusion protein consisting of the GAL4 DNA-binding domain fused to the activation domain of NFκB. These effector cells were mixed with a separate set of reporter cells that were transfected with a reporter plasmid containing the luciferase gene under the control of a GAL4 responsive promoter. HRSV F mediated cell fusion of the two cell populations results in co-localization of the GAL4-NFκB transactivator fusion protein with the GAL4 responsive luciferase reporter plasmid and subsequent transcriptional transactivation of the reporter gene. A dilution series from 50,000 to 100 effector cells were added to a fixed amount of reporter cells (50,000), and luciferase activity was monitored 24 hours later. As a control to determine the maximum signal, cells were co-transfected with the reporter and activator plasmids (pFR-Luc + pBD-NFκB). As shown in figure 2A, luciferase activity was absent in cells transfected with either reporter or activator plasmids alone. Additionally, mixing of cells which had been separately transfected with the reporter or activator plasmids did not produce detectable luciferase activity indicating no spontaneous cell fusion (data not shown). However, mixing an increasing number of cells that had been co-transfected with the GAL4-sensitive reporter plasmid and HRSV F expressing plasmid with those that had been transfected with the GAL-NFκB activator plasmid resulted in a dose-dependent increase in luciferase activity (Figure 2A), indicating fusion of the two cell populations. The maximum signal observed from mixing the effector and reporter populations was similar to the signal obtained when the activator and reporter plasmids were co-transfected into the same cells.

**Figure 3:**
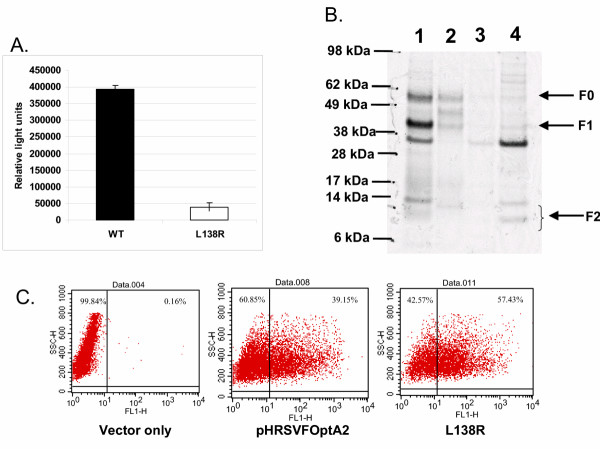
A) Comparison of fusion activity of wild-type and a fusion peptide mutant of HRSV F. 293T cells co-transfected with pBD-NFκB and either pHRSVFOptA2 or pL138R were mixed 24 hours later with a separate population of 293T cells that had been transfected with pFR-Luc, and luciferase activity was measured 24 hours post mixing as described in methods. Luciferase activity is reported as relative light units. B) Processing of the wild-type and L138R mutant of HRSV F was determined by metabolic labeling 293T cells transfected with either pHRSVFOptA2 (lane 1), pL138R (lane 2), pCMV-β-gal (lane 3), or mock transfected (lane 4) for 6 hours with [^35^S]-cysteine/methionine followed by immunoprecipitation of lysates with HRSV F specific mAbs, and analysis of immunoprecipitates by SDS-PAGE as described in methods. C) Cell surface expression of the wild-type and L138R mutant F proteins in 293T cells transfected with either pHRSVFOptA2 or pL138R was compared by flow cytometry as described in methods.

To determine if this property was restricted to the F protein derived from a single strain or subgroup, 293T cells co-transfected with a plasmid encoding the HRSV F protein derived from either subgroup A (A2 strain) or B (18537 strain) together with a plasmid encoding the GAL4-NFκB transactivator fusion protein (effector cells) were then mixed 24 hours later with an equal amount of a separate population of 293T cells (reporter cells) which had been transfected with the GAL4 responsive reporter plasmid. As shown in Figure 2B, the F protein of either HRSV subgroup A and B mediated cell-cell fusion as measured by the increased luciferase activity relative to the negative control (GAL4-NFκB transactivator fusion protein only). The fusion activity of the F protein derived from the A2 strain was approximately 2-fold higher than that observed with the 18537 strain despite similar expression levels. Whether this finding reflects differences in the pathogenicity between these two isolates is unknown, although a recent study suggests similar pathogenicity for both subgroups [35]. To further confirm that the observed fusion activity was inherent to the HRSV F protein, a point mutation (L138R) was generated in the fusion peptide consensus sequence within the fusion peptide region. Mutation of leucine residue 138 to arginine reduced fusion activity to 10% relative to wild-type (Figure 3A). Despite the fact that this mutant appeared to produce somewhat lower levels of fully processed F protein (Figure 3B, lane 2) for unknown reasons, this mutant was expressed at high levels on the cell surface (Figure 3C) indicating that the cell fusion observed was attributable to the HRSV F protein.

### Host range of HRSV-mediated fusion

To determine the host range of HRSV F mediated fusion using the quantitative fusion assay. 293T effector cells were prepared as described above. As we previously demonstrated proper protein processing, abundant cell surface expression of HRSV F protein and cell fusion activity using 293T cells, we selected these as our effector cells. These effector cells were mixed with reporter cells derived from a diverse range of species (Table 1) that were transfected with the GAL4-responsive reporter plasmid. To account for any differences in relative transfection efficiencies and expression of the luciferase reporter among the various target cells lines, the target cell lines were transfected with a plasmid containing the luciferase gene under the control of the SV40 early promoter (pGL3-control), and relative luciferase activity was measured. To account for potential differences in host cell transcription factors that mediate activation of the reporter plasmid, the assay was flipped and 293T cells were co-transfected with the HRSV F expression plasmid and the GAL4 responsive reporter plasmid, and the cells from the various species were transfected with the GAL4-NFκB transactivator fusion protein plasmid. For further comparison, we used the VSV G protein, which is known to mediate entry into cells derived from a wide range of species.

**Table 1:** Species and tissue origin of cell lines used in this study are listed.

**Cell line**	**Species, tissue**
XLK-WG	*Xenopus laevis *(S. African clawed frog), kidney
QT6	*Coturnix coturnix japonica *(Japanese quail), fibrosarcoma
Tb1Lu	*Tadarida brasiliensis *(free-tailed bat), lung
NIH/3T3	*Mus musculus *(mouse), fibroblast
BHK-21	*Mesocricetus auratus *(Syrian golden hamster), kidney
RK-13	*Oryctolagus cuniculus *(rabbit), kidney
LLC-PK1	*Sus scrofa *(pig), kidney
Mv1Lu	*Musteal vison *(mink), lung
AK-D	*Felis catus *(domestic cat), fetal epithelial
MDCK	*Canis familiaris *(domestic dog), kidney
MDBK	*Bos taurus *(cow), kidney
E. Derm	*Equus caballus *(horse), dermal
Vero	*Cercopithecus aethiops *(African green monkey), kidney
HEp-2	*Homo sapiens *(human), laryngeal carcinoma
HeLa	*Homo sapiens *(human), cervical carcinoma
MT-4	*Homo sapiens *(human), T-cell
293T	*Homo sapiens *(human), kidney
NCI-H292	*Homo sapiens *(human), epidermoid pulmonary carcinoma
A549	*Homo sapiens *(human), lung

**Figure 4:**
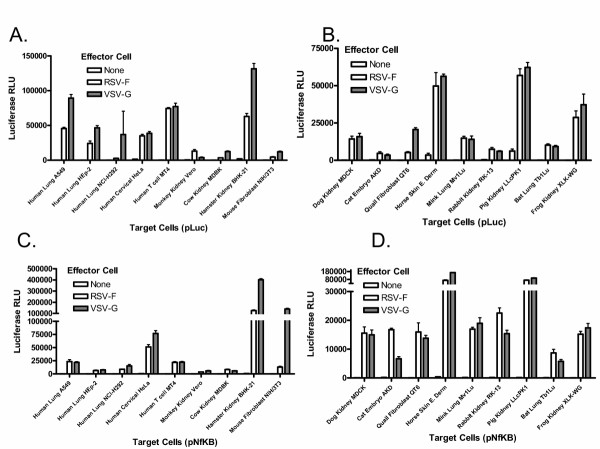
Fusion activity of HRSV F with cell lines derived from various species. Cell lines derived from various species (target cells) were either transfected with pFR-Luc and mixed 24 hours later with 293T cells that had been co-transfected for 24 hours with pHRSVFOptA2 or pVPack-VSV-G and pBD-NFκB (Figs. 4A and 4B), or the target cells were transfected with pBD-NFκB and mixed 24 hours later with 293T cells that had been co-transfected for 24 hours with pHRSVFOptA2 or pVPack-VSV-G together with pFR-Luc (Figs. 4C and 4D). Cell lines derived from various species were transfected with either pFR-Luc or pBD-NFκB only as negative controls. Luciferase activity was measured 24 hours post mixing of the cell populations as described in methods and is reported as relative light units.

As shown in figure 4, despite a limited host range in nature, HRSV F was able to mediate fusion to various degrees with cells derived from all species examined. This fusion activity was within 5-fold of the fusion activity mediated by the VSV G protein in the cell types tested here. Generally, there was little qualitative difference between results obtained when either the reporter plasmid or the activator plasmid were co-transfected with the F expression plasmid (compare figures 4A and 4B with figures 4C and 4D). As expected, the relative transfection efficiencies of the various cell lines as measured by the luciferase activity from the plasmid pGL3-control varied; however, there was no direct correlation between transfection efficiencies and fusion activity. For example, cell lines such as BHK-21 and LLC-PK1 cells which transfected well, had lower relative levels of fusion. In contrast, cell lines such as MT-4, MDCK and XLK-WG which had low transfection efficiency, had appreciable levels of HRSV F mediated fusion. These findings support the hypothesis that HRSV F protein interacts with evolutionarily conserved host cell surface molecules or can use multiple mechanisms to enter cells.

### Infections using recombinant HRSV expressing GFP

**Figure 5:**
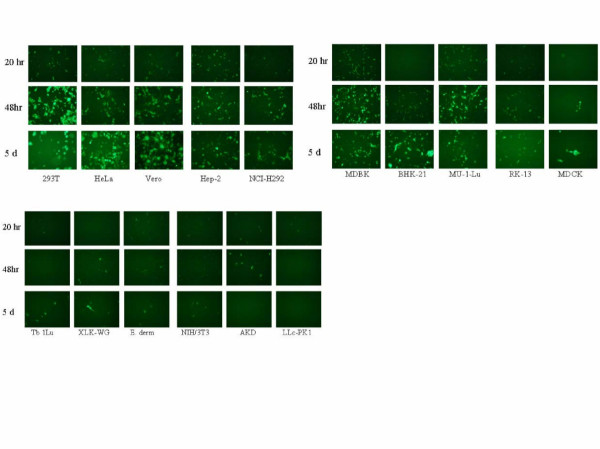
Infection of various cell lines by rg224(RSV). Cell lines derived from various species were infected with rgRSV(224) at an MOI = 0.1 and GFP-expressing cells were visualized at 20, 48, and 120 hours post infection by fluorescent microscopy by monitoring fluorescence at 488 nm.

The results obtained from the fusion assays indicated that HRSV F is able to mediate fusion with cells from multiple diverse species, suggesting that virus entry is not the primary determinant of host range. To examine whether viral mRNA transcription had occurred, the various cell lines were infected with a recombinant HRSV (rgRSV224) expressing GFP [33] and fluorescence scored at 20, 48, and 120 hours post infection. As expected, rgRSV(224) infection of human (HEp-2, HeLa, A549) and animal (Vero, Mv1Lu, MDBK) [36-39] cell lines commonly used to propagate HRSV resulted in a time dependent increase in the number of cells expressing GFP (≥50% by day 5) as seen by fluorescent microscopy indicating spread of infection throughout the culture (Figure 5). Infection of other human cell lines such as NCI-H292 [40], and 293T also resulted in a time dependent increase in the number of cells expressing GFP. Infection of hamster BHK-21 cells also resulted in a time dependent increase in the number of GFP positive cells, although the appearance of a large number of bright GFP positive cells seemed delayed. Interestingly, hamsters are considered to be a semi-permissive host for HRSV [27,41] and produce lung titers similar to those achieved in mice. Whether this reflects a tissue-specific phenomenon (kidney versus lung) remains to be determined. Infection of cell lines (Tb1Lu, AK-D, E. Derm, NIH/3T3, LLC-PK1, and XLG-WG) derived from other species (bat, cat, horse, mouse, and frog respectively) produced few or occasional GFP expressing cells over the course of the five-day infection. The number of positive cells did not increase over time, and in some cases (AK-D cells) appeared to decrease. Aside from mice, infection in vivo of these other species by HRSV has not been described. This finding also supports the finding that high titers of virus (>10^5 ^PFU) are typically required to initiate infection in mice after intranasal inoculation, and that relatively few cells become viral antigen positive.

## Discussion

We have developed a quantitative reporter gene based cell-cell fusion assay for HRSV F. Prior assays have been based upon visual counting of plaques or syncytia after staining of infected monolayers, or infection another virus such as vaccinia, to provide HRSV F protein, which could potentially complicate interpretation. The assay described herein is a means of quantifying the fusion activity of the HRSV F protein. This assay has multiple applications. For example, this assay can be used as a means of studying the structure-function of the HRSV F protein, or for evaluating the activity of mutations in the F protein without the need to select for antibody or compound escape mutants or generate point mutations in a reverse genetics system. We propose that this assay also has utility in the identification and characterization of inhibitors of HRSV entry for the development of specific agents to prevent and treat HRSV infections. We have used this assay as a means of exploring the host-range of HRSV and have shown that the HRSV F protein is able to mediate fusion with cells derived from a wide range of vertebrate species.

Cell lines known to be permissive for HRSV growth such as HEp-2, HeLa, A549, Vero, MDBK, and Mv1Lu were highly competent for F protein fusion as expected. Somewhat surprisingly, a wide variety of cells derived from species not known to be normally infected by HRSV were also capable of undergoing HRSV F protein mediated fusion. Most surprising were the results obtained using the XLK-WG cells which are derived from the amphibian *Xenopus laevis*. Although this finding implies that HRSV virion is able to enter a wide range of cells, the results of the infection studies using the GFP-expressing RSV indicate that viral mRNA transcription seems limited in cell lines derived from certain species. Taken together these results suggest that events post-viral entry are the primary determinants that mediate the host range of HRSV. During natural infection of humans, viral replication is restricted to epithelial cells of the upper and lower respiratory tract. Although limited HRSV replication within human alveolar macrophages and detection of HRSV sequences in peripheral blood monocytes (PBMCs) has also been reported [42,43], dissemination of HRSV to other organs is not observed even in immunocompromised individuals. Similarly, disseminated infection with bovine RSV is not observed in infected cattle [44]. Given the ability of the HRSV F protein to mediate fusion with cells derived from a diverse range of vertebrate species, the implication is that HRSV may not be able to access these sites or undergoes non-productive infection in many cell types other than epithelial cells of the respiratory tract. Although the overall biological significance of such an abortive infection is unclear, biological effects of the individual HRSV proteins have been reported.

HRSV F protein has also been shown to be a ligand for TLR4, and HRSV infection persists longer in TLR4-/- deficient mice [45,46]. HRSV F protein also binds surfactant proteins A and D (SP-A and SP-D) [47,48], although the implications of these findings in human infection are unclear. G protein has been shown to modulate multiple immune related activities. Soluble G suppresses some PBMC and lung CD8+ T-cell effector and peripheral memory responses [49], induces chemotaxis, eosinophilia, and both soluble and membrane forms of G bind the fractalkine receptor, CX3CRI [50]. Additionally, G has a domain with similarity to the TNF-α receptor (p55), although it has not been directly shown to be a TNFR antagonist. Additionally, G has been shown to modify CC and CXC chemokine mRNA expression [50], and suppress lymphoproliferative responses to antigens in PBMCs [51].

It is tempting to speculate that entry of HRSV into cell types other than those permissive for complete virus growth may be a strategy by which the virus is able to modulate immune responses while avoiding the induction of antiviral responses such as the interferon (IFN) pathway by production of double-stranded RNA replication intermediates in these cells. Limited viral mRNA transcription in the absence of virus RNA replication would result in expression of NS1 and NS2 which have been shown to block the IFN response [52] possibly preventing these unproductively infected cells from responding to external cytokines such as IFNs. Such a strategy may help explain why despite little antigenic drift in the F protein, infection by HRSV infection only confers partial protection, with reinfections occurring throughout life [53-55]. As the fusion proteins of other members of the *Paramyxoviridae *family, such as Hendra virus [56], are also able to mediate fusion with a wide variety of cells derived from multiple species, it is possible that such a strategy is shared by other members of this virus family.

## Competing interests

The author(s) declare that they are all employees of Centocor, Inc. which provided supported for this work.

## Authors' contributions

PB and CL contributed equally to this work. PB and ND performed the fusion assays, immunoprecipitations, and flow cytometry. CL generated reagents and developed the fusion assay. LG conducted site-directed mutagenesis of the HRSV F protein. AD and RS participated in the design of the experiments, oversight of the conduct of the experiments, and in the interpretation of the results.

**Table 2:** Infection of various cell lines with GFP-expressing HRSV.

**Cell line**	**20 hrs**	**48 hrs**	**120 hrs**
Vero	+++	++++	++++
AK-D	+	++	-
MDBK	+++	++++	+++
MDCK	+	+	+
Tb1Lu	+	+	+
XLK-WG	-	+	+
E. Derm	++	++	+
HeLa	++	+++	++++
NCI-H292	++	+++	+++
293T	++	++++	++++ *
HEp-2	+++	++++	++++
Mv1Lu	+++	++++	+++
NIH/3T3	+	+	+
LLC-PK1	+	-	-
RK-13	++	++	++
BHK-21	-	++	+++
QT6	+	++	++++
A549	+	+++	++
MT-4	+	++	++

- = few isolated weakly positive cells

+ = <1–5% GFP positive cells in culture

++ = 5–30% GFP positive cells in culture

+++ = 30–60% GFP positive cells in culture

++++ = >60% GFP positive cells in culture

* wide spread cell death
